# Time-, Location-, and Layer-Specific Chorioretinal Biomarkers in a Translational Large-Animal Porcine Model of Chorioretinal Ischemia

**DOI:** 10.1167/iovs.67.12.8

**Published:** 2026-10-05

**Authors:** Niklas Junker, Noel Josua Olfert, Ludwig Geisweid, Anna Stoß, Bryan Calder Ackermann, Laura Ruddeck, Jakob Michael Rödl, Martin Stephan Spitzer, Gerd Uwe Auffarth, Victor Aristide Augustin, Maximilian Hammer

**Affiliations:** 1 David J. Apple Laboratory for Vision Research, Heidelberg, Germany; 2 Department of Ophthalmology, University Hospital Heidelberg, Heidelberg, Germany; 3 Department of Ophthalmology, University Hospital Hamburg Eppendorf, Hamburg, Germany

**Keywords:** chorioretinal ischemia, CRAO, OCT, biomarkers, porcine model

## Abstract

**Purpose:**

Conditions associated with complete or partial ocular ischemia, such as central retinal artery occlusion (CRAO), are vision-threatening emergencies. Therapeutic interventions are highly time-dependent, yet the exact onset of ischemia is often uncertain. Optical coherence tomography (OCT) has emerged as a potential tool for objective staging; however, quantitative, layer-resolved OCT data within the earliest phase of retinal ischemia in clinically relevant large-animal models are lacking.

**Methods:**

Ten eyes from 10 pigs were included. Physiological retinal perfusion was confirmed using fluorescein-, indocyanine-green-, and OCT angiography (OCTA). Baseline chorioretinal morphology was assessed by OCT. Global retinal ischemia was induced by controlled cardiac arrest. Electrophysiological response of the retina after ischemia was measured using electroretinography (ERG) and serial OCT imaging was performed at intervals of 2.5 to 30 minutes for up to 270 minutes postmortem. Layer- and region-specific changes were quantified using dedicated image analysis software.

**Results:**

ERG response was abolished within 2 minutes after cardiac arrest and structural retinal changes were immediately detectable and followed a near-linear progression over time. Total retinal thickness increased approximately 1.56-fold within 270 minutes. Layer-specific swelling differed markedly, ranging from 1.5-fold in the outer nuclear layer to 2.2-fold in the inner plexiform layer. Despite these differences, swelling kinetics were remarkably conserved across retinal regions.

**Conclusions:**

Layer-specific OCT changes occur within minutes after retinal ischemia and exhibit highly reproducible, near-linear kinetics independent of retinal location. These findings establish a quantitative framework for time-resolved staging of retinal ischemia and may enable objective estimation of ischemia onset in clinical settings.

Diseases accompanied by acute ocular ischemia are vision-threatening emergencies on the rise, due to the increasing incidence of cardiovascular risk factors and the aging population.^1^ One of the most feared among these conditions is central artery occlusion (CRAO), causing complete interruption of the retinal blood circulation followed by an ischemia of the inner retinal layers. Because the retina is a neuronal tissue, this leads to substantial dysfunction and a pronounced vision loss in the affected eye.^2^^,^^3^ Furthermore, CRAO is considered a subtype of ischemic stroke requiring immediate neurological care.^4^

Addressing this severe ischemic retinal disease therapeutically remains a major unresolved challenge. Multiple strategies have been explored in an attempt to improve visual outcomes in CRAO,^5^ including systemic vasodilators,^6^ ocular pressure reduction,^7^^,^^8^ laser-based embolectomy,^9^ hyperbaric oxygen therapy (HBOT),^10^ and transdermal electrostimulation,^11^ as well as local and systemic fibrinolysis.^12^^,^^13^ None of these approaches has demonstrated consistent or reproducible benefit in improving visual acuity, and no evidence-based treatment currently exists for CRAO. Whereas local intra-arterial fibrinolysis was the first treatment to be investigated in a randomized controlled trial within the EAGLE trial,^13^ systemic thrombolysis, which had shown early promise in observational studies,^12^ was the most extensively investigated intervention in recent years. However, results from recent randomized controlled trials have failed to demonstrate a statistically significant visual benefit of early thrombolysis over standard care.^14^^,^^15^ Most recently, the German REVISION trial was terminated after showing no beneficial effect in an interim analysis with publication of the results still pending.

Irrespective of the therapeutic approach, determining the time of ischemia is crucial for the assessment of possible therapeutic options, the visual outcome after therapy, as well as further research,^1^^,^^3^^,^^12^ especially because previous basic research data suggested a certain degree of tolerance of the retina to ischemia.^3^^,^^16^ However, the onset of the symptoms is often insufficiently remembered by the patient complicating clinical decision making, risk stratification, and outcome prediction. Optical coherence tomography (OCT) provides a powerful, noninvasive diagnostic tool to assess morphological changes in the retina of patients with CRAO.^17^^–^^19^ Especially quantification of retinal swelling via OCT has previously been identified as a valuable method to objectively determine ischemia onset.^20^^–^^22^ However, comprehensive data on the temporal, layer-specific, and spatial characteristics of post-ischemic retinal changes are still lacking. This knowledge is particularly important for future translational approaches, the establishment of OCT-biomarkers with granular temporal resolution, and enhancement of animal models for chorioretinal ischemia.

In this study, we therefore aimed to thoroughly characterize the changes in retinal morphology in untreated porcine eyes confirmed healthy by means of fluorescein-, indocyanine-green-, and OCT angiography (OCTA). After gathering baseline data on the retinal and choroidal ultrastructure in the living animals, structural biomarkers were evaluated in situ for 4.5 hours after onset of chorioretinal ischemia using OCT. Using image analysis, the swelling behavior was specifically characterized time-, location-, and layer-wise.

## Methods

### Animals

For this study, 10 left eyes of 10 pigs were examined. The eyes included in this study served as control eyes for a 6-week biocompatibility study in the fellow right eyes of the animals. The eyes included in this study were untreated and their health status was ensured using a multimodal ophthalmic examination consisting of electroretinography (ERG), OCT, OCTA, and measurement of intraocular pressure (Supplementary Fig. S1). The animals were housed in the Interfaculty Biomedical Research Facility (IBF, Heidelberg, Germany) with restricted food access and unrestricted access to water. All interventions on the animals were approved by the relevant animal welfare authority (approval number: 35-9185.81/G-11/24, Regierungspräsidium Karlsruhe) and aligned with the ARVO guidelines for the use of animals in ophthalmic and vision research and the ARRIVE guidelines, as well as German animal welfare laws and relevant directives of the European Union (2010/63/EU).

### Anesthesia and Induction of Chorioretinal Ischemia

For the baseline OCT scans and pre-experimental angiography, the animals were sedated with azaperon (6 mg/kg; WDT, Garbsen, Germany) by intramuscular injection. After sedation, anesthesia was induced with intramuscular injection of ketamine (11 mg/kg; CP Pharma, Burgdorf, Germany) and midazolam (2 mg/kg; Hameln Pharma, Hameln, Germany) and maintained with isoflurane (2% vol/vol; CP Pharma, Burgdorf, Germany).

Chorioretinal ischemia was induced by euthanization of the animals via intravenous injection of 60 mL potassium chloride 7.45% solution (B. Braun, Melsungen, Germany) after further deepening of the anesthesia with Propofol 10 mg/mL (CP Pharma). Cardiac arrest was confirmed via measurement of vital parameters and absence of heart sounds detected on auscultation.

After cardiac arrest, the animals were kept warm using a mobile heating device under monitoring of body temperature to imitate physiological body temperature after death. The cornea of the animals was treated with carbomer 2 mg/g (Dr. Gerhard Mann Pharma, Berlin, Germany) to avoid corneal drying and to ensure appropriate visualization of the posterior pole for OCT scans after death.

### Angiographic Examinations

For fluoresceine and indocyanine-green angiography (FA/ICGA), a mixture of 0.5 mL of 10% fluorescein (Alcon, Freiburg, Germany) and 0.5 mL of 5 mg/mL of indocyanine green (Mediconsult, Roggwil, Switzerland) was injected in the auricular vein of the animals as a bolus. After injection, images were captured with the Heidelberg Spectralis Flex System (Heidelberg Engineering, Heidelberg, Germany) in the Blue-Autofluorescence/Infrared-Autofluorescence (BAF/IRAF) mode for up to 10 minutes post-injection.

For OCTA, a 10-degree scan located in the visual streak of the animals was acquired using the Heidelberg Spectralis Flex System (Heidelberg Engineering, Heidelberg, Germany) with 7 frames per scan. En face images of the superficial vascular complex (SVC) and deep vascular complex (DVC) were exported after manual confirmation of the correct identification of the retinal layers by the custom stratification algorithm of the Heyex software.

### Electroretinography

ERG testing was performed immediately prior to induction of ischemia, as well es repeatedly after cardiac arrest until the first postmortem OCT examination was performed 2.5 minutes after death. The tests were conducted using the RETevet system (LKC Technologies, Gaithersburg, MD, USA). Because dark adaption would have interfered with the prioritized OCT scan acquisition and the imaging timetable, only photopic ERG tests were performed according to the Dog, Cat, Nonhuman Primate International Society for Clinical Electrophysiology of Vision (ISCEV) 6 Step testing procedure. The testing protocols are presented in Supplementary Table S1.

### Optical Coherence Tomography

Retinal ultrastructure after induction of chorioretinal ischemia was assessed using OCT. Therefore, a high-resolution volume scan grid consisting of 49 horizontal B-scans spanning 5.6 × 5.6 mm were placed on the porcine eyes posterior pole with the central B-scan (25/49) located midway between the superior and the inferior vascular arcade. The superior margin of the Dense scan was hereby located near the superior vascular arcade and the inferior margin was located near the inferior vascular arcade of the pig’s posterior pole. Each individual B-scan contained 512 A-scans with an automated real-time (ART) averaging of 58 frames.

Repeated OCT scans were performed using the follow-up function of the Heidelberg Spectralis System 2.5, 5, 7.5, 10, 12.5, 15, 20, 25, 35, 45, and 60 minutes after induction of chorioretinal ischemia, as well as every 30 minutes after 60 minutes for up to 270 minutes postmortem. The experimental setup is displayed in Figure 1.

**Figure 1.**
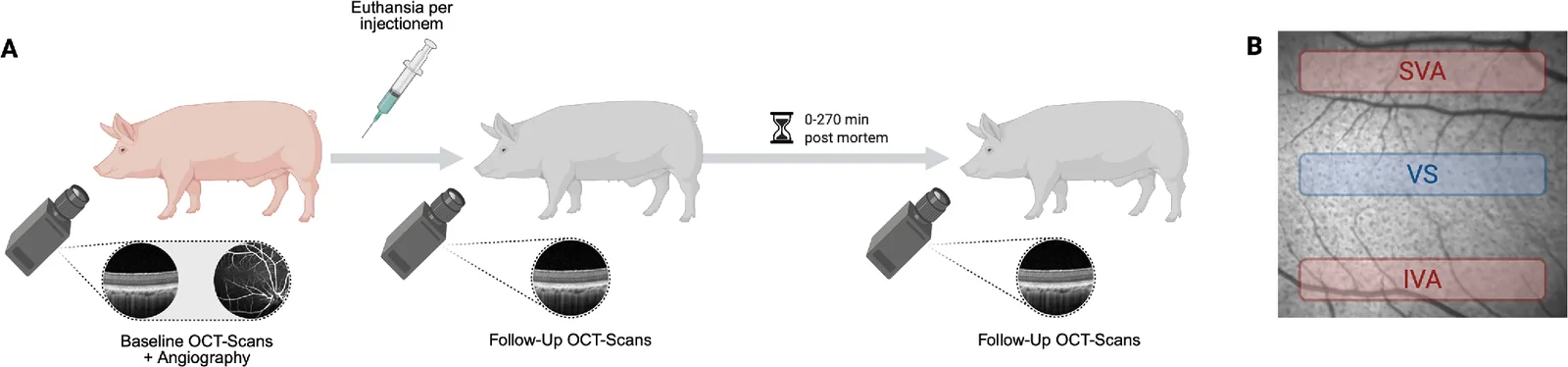
**Experimental setup of the study.** (**A**) Ten German Landrace pigs received baseline OCT scans on their left eye. To ensure physiological retinal and choroidal vascularization, the animals additionally underwent angiographic examinations. Full chorioretinal ischemia was induced by euthanization of the animals via injection. Follow-up OCT scans were performed at different time points up to 270 minutes postmortem. The OCT scans were exported and retinal layers were quantified using image processing software. (**B**) OCT scans from different retinal regions including the central visual streak region and the regions around the vascular arcades were included in the study. IVA, inferior vascular arcade; OCT, optical coherence tomography; SVA, superior vascular arcade; VS, visual streak.

### Image Analysis

Images were exported from the Heidelberg Eye Explorer (HEYEX, version: 2.6.6) for offline analysis. Only such B-scans were included in the analysis, for which the internal image quality score (Q-score) of the Heyex software was ≥ 20 across all time points.

Retinal layers were manually measured using Fiji/ImageJ software (version 1.54p) by a blinded investigator (author N.O.). Scaling was based on the embedded 1:1 µm OCT metadata, whereas layer boundaries were defined according to the IN-OCT Consensus nomenclature.^23^

Five B-scans were selected in total from each volume scan to represent the visual streak, as well as the inferior and superior regions. One B-scan was selected from the inferior region (scans 1–10), three from the visual streak (scans 15–35), and one from the superior region (scans 40–49; see the Table). These individually predefined scan locations were then used at all time points within each animal to ensure longitudinal comparability.

**Table.** Number of Included Animals and Scans Per Location

Location	Number of Animals	Number of Scans Per Animal
Superior vascular arcade (SUP)	10	1
Visual streak (VS)	10	3
Inferior vascular arcade (INF)	10	1

The following retinal layers were measured in the same predefined order across all examinations: retinal nerve fiber layer (RNFL), ganglion cell layer (GCL), inner plexiform layer (IPL), inner nuclear layer (INL), outer nuclear layer (ONL), and total retinal thickness (TRT). Outer plexiform layer (OPL), photoreceptor (PR) layer, and retinal pigment epithelium (RPE) was not quantified due to their low thickness and different discrimination to adjacent layers. Their thickness was therefore analyzed together as OPL + PR + RPE calculated as the difference between TRT and the sum of all other layers.

To ensure measurements were performed perpendicular to the RPE, the Angle tool in Fiji (ImageJ software) was used, with local retinal curvature taken into account.

### Statistical Analysis

Statistical analysis was performed using GraphPad Prism (version 11; GraphPad Software, San Diego, CA, USA). Mean thickness, standard deviation, and parts of whole were calculated for the scans in the visual streak area, near the superior vascular arcade and near the inferior vascular arcade separately. Linear regression analysis was performed on the range of data demonstrating linear behavior as determined by visual inspection. Relative swelling was calculated relative to baseline thickness of the retinal layers across all regions. Thickness of the retinal layers and the whole retina at baseline, as well as relative swelling after 270 minutes, was compared using Student's *t*-test.

## Results

### Angiographic Examinations Prior to Ischemia Revealed Physiological Chorioretinal Perfusion

To confirm physiological chorioretinal macro- and microvasculature in the studied animals, FA-ICGA, as well as OCTA were performed prior to the experiment. FA revealed an unimpaired filling of the retinal vessels in choroidal, arterial, arteriovenous, and venous phase in all animals. No areas of malperfusion or abnormal vessel configuration, as well as no signs of leakage or neovascularization occurred (see Supplementary Fig. S1A). In ICGA, a physiological conformation of the choroidal vasculature and a homogenous choroidal perfusion of the posterior pole was observed (see Supplementary Fig. S1B). Examination of the SVC (see Supplementary Fig. S1C) and the DVC (see Supplementary Fig. S1D) in OCTA presented an intact microvasculature.

The examined animals were therefore suited for the followed OCT-based evaluation of the retinal morphology after chorioretinal ischemia.

### Changes in Electrophysiological Response and Chorioretinal Morphology Commenced Immediately After Induction of Ischemia

Immediately after the induction of chorioretinal ischemia, a process of rapid and continuous decline in electrophysiological response of the retina started, as evidenced by ERG testing. In both conducted light-adapted tests, amplitudes decreased and latencies increased constantly and ERG response was completely abolished within 2.5 minutes postmortem (Figs. 2A, 2B).

**Figure 2.**
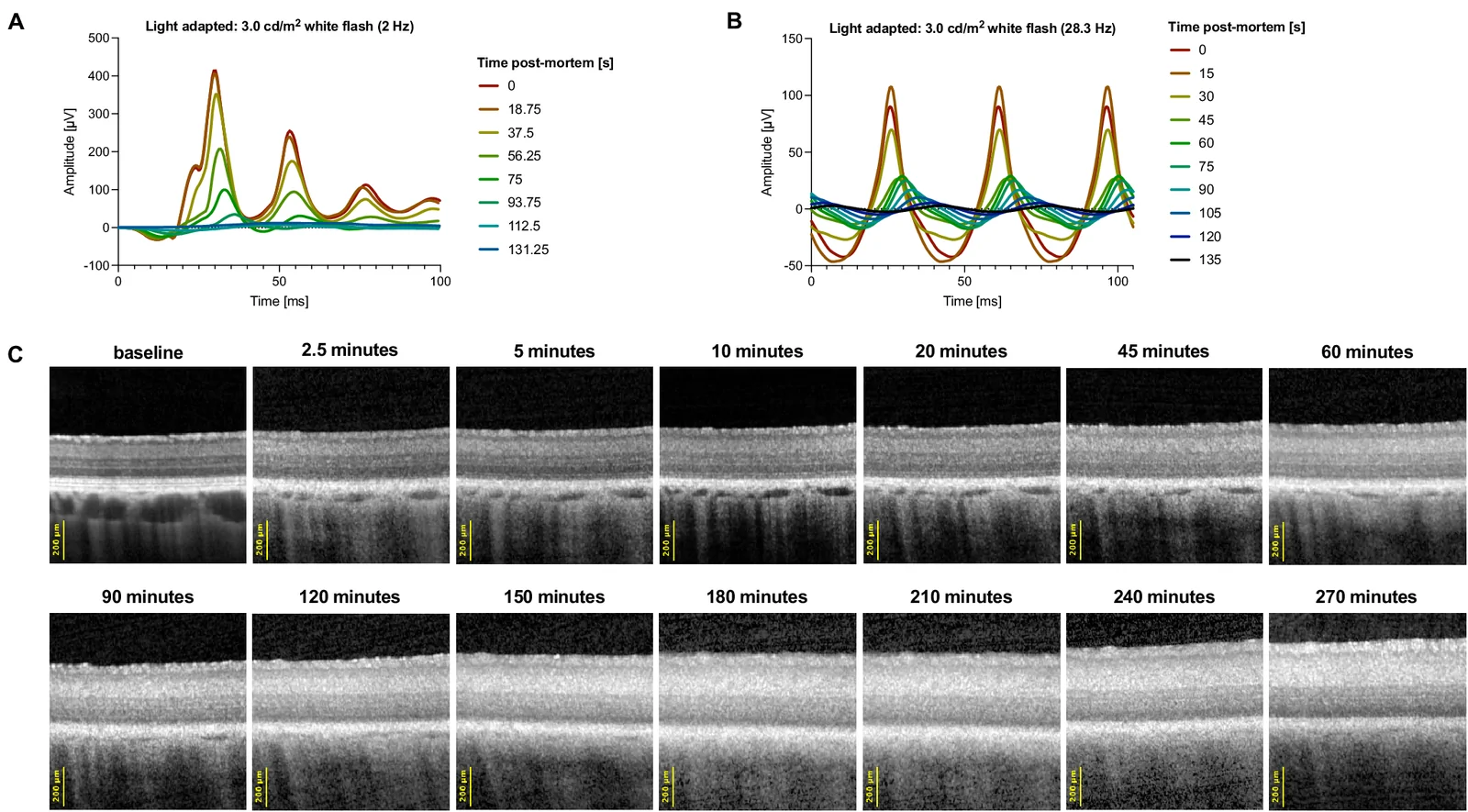
**Electrophysiological and morphological changes to the retina after induction of chorioretinal ischemia.** The electrophysiological response of the retina showed a rapid decline within the first minutes after induction of ischemia in two light-adapted ERG tests (**A,** **B**). Even before acquisition of the first OCT scan 2.5 minutes postmortem, the retinal response to light stimuli was completely abolished. (**C**) The first changes in chorioretinal morphology appeared immediately after induction of ischemia with the collapse of the choroidea and blurring of the retinal layers. Over the course of the study, incremental retinal swelling occurred with the IPL showing the most remarkable increase in thickness. After 270 minutes post ischemia, the retina presented in an edematous, hyper-reflective state. ERG data and OCT images are shown for representative animals. ERG, electroretinography.

Morphological changes in the porcine choroidea and retina began to occur immediately after chorioretinal ischemia. Most specifically, an immediate collapse of the choroidea was observed, as well as blurring of the retinal layering. The typical discrimination between the ellipsoid zone (EZ) and the interdigitation zone (IDZ) of the photoreceptors outer segments was immediately abolished (Fig. 2C, 2.5 minutes). Over the course of the ischemia, an incremental process of swelling occurred, with the IPL exhibiting the most remarkable increase of thickness (see Fig. 2C, 60 minutes). Furthermore, the retinal layers, became more reflective, specifically the IPL. After 270 minutes, the thickness of the whole retina had significantly increased and presented in a hyper-reflective, edematous state with blurred borders of the retinal layers (see Fig. 2C, 270 minutes).

### Retinal Layers Showed a Reproducible and Distinct Swelling With Increasing Ischemia

TRT increased in the visual streak region (VS) from 265 ± 24 (mean ± SD) µm at baseline to 407 ± 30 µm after 270 minutes (Fig. 3A). Thickness of RNFL, GCL, and IPL increased from 32 ± 4 µm (mean ± SD), 21 ± 2 µm, and 51 ± 7 µm to 58 ± 6 µm, 46 ± 7 µm, and 110 ± 11 µm, respectively, during the 4.5 hours of ischemia (Figs. 3B, 3C, 3G). INL and ONL swelled from 20 ± 4 µm and 43 ± 7 µm to 35 ± 9 µm and 64 ± 9 µm over the course of the study (Figs. 3H, 3I). Descriptive statistics of the thickness increase in the three different regions is presented in Supplementary Table S2.

**Figure 3.**
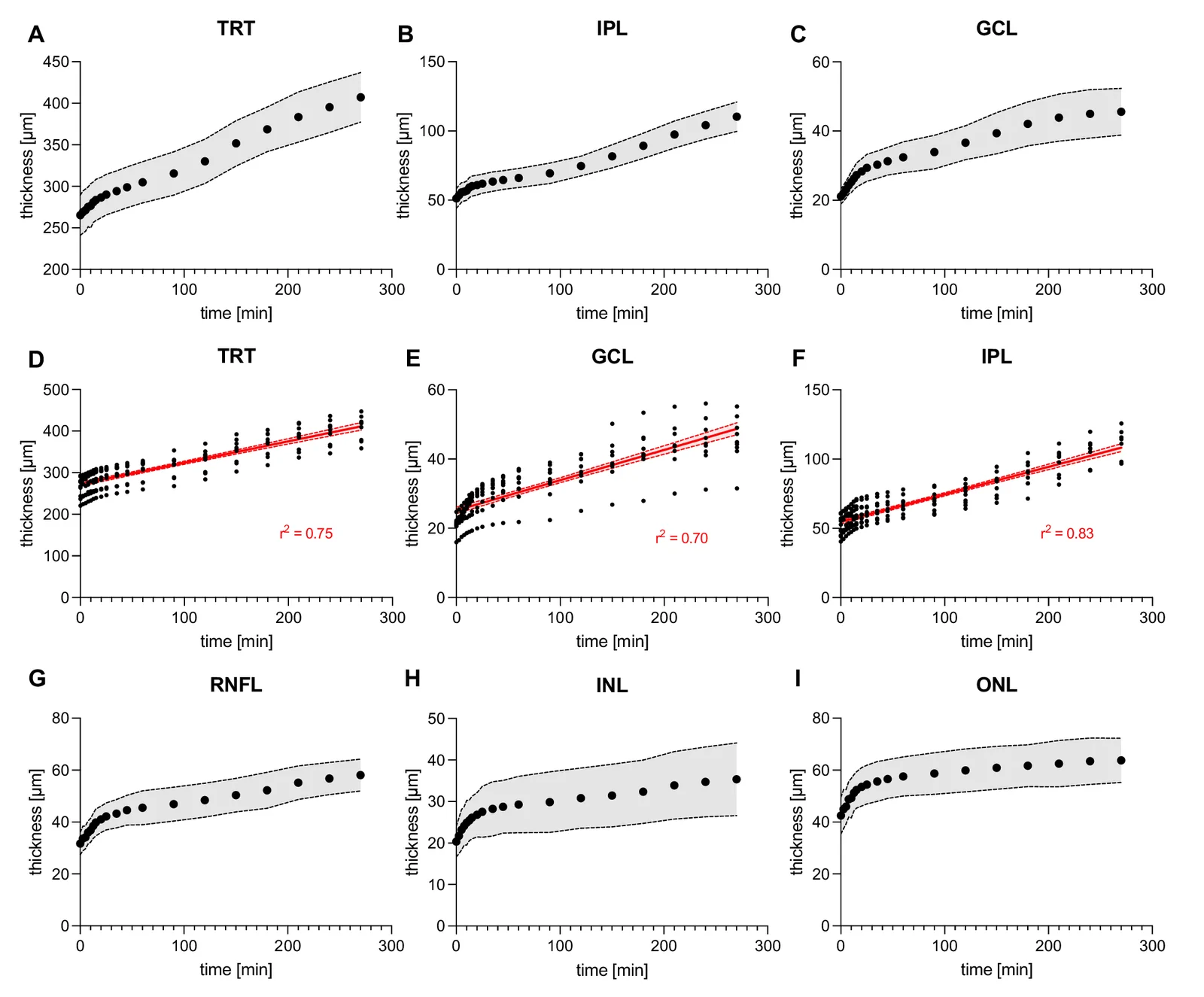
**Increase in thickness of the retina and its different layers during ischemia.** Measurement of TRT, as well as thickness of the respective layers revealed an incremental swelling process over the course of the study. Thickness of the whole retina (TRT) (**A,** **D**), IPL (**B,** **E**), and GCL (**C,** **F**) increased in a linear manner after approximately 45 to 60 minutes of nonlinear growth. RNFL (**G**), INL (**H**), and ONL (**I**) presented, after a rapid thickness increase in the first minutes of ischemia, a slow, nonlinear swelling process. Mean and standard deviation (*light grey* area) is shown. The *light red* area indicated the 95% confidence interval of the respective linear regression. GCL, ganglion cell layer; IPL, inner plexiform layer; INL, inner nuclear layer; ONL, outer nuclear layer; RNFL, retinal nerve fiber layer; TRT, total retinal thickness.

### Total Retinal Thickness, Ganglion Cell Layer, and Inner Plexiform Layer Showed a Reproducible Near-Linear Increase in Thickness

Analyzing the specific swelling behavior of the different retinal layers in the VS, thickness of TRT, GCL, and IPL increased linearly after about 45 minutes over the course of the following 225 minutes. TRT exhibited a swelling rate of 0.51 µm/min (95% confidence interval [CI] = 0.46–0.56, *P* < 0.0001, *r*^2^ = 0.75; Fig. 3D), whereas GCL swelling was 0.09 µm/min (95% CI = 0.08–0.1, *P* < 0.0001, *r*^2^ = 0.71; Fig. 3E) and IPL swelling was 0.20 µm/min (95% CI = 0.18–0.21, *P* < 0.001, *r*^2^ = 0.83; Fig. 3F).

### Retinal Layers Show Distinct Relative Swelling Rates

In the VS, the retina swelled to 1.56 ± 0.05 times its initial thickness after 270 minutes (see Fig. 3A, Fig. 4A). When comparing the composition of the TRT at baseline and after 270 minutes, markedly altered proportions of the individual layers contributing to the overall thickness are observed (see Figs. 4A, 4B). This was most pronounced for the IPL: at baseline, IPL thickness contributed to 19.3 ± 1.5% of TRT, a proportion that rose to 27.0 ± 2.1% after 270 minutes. For the other retinal layers, this proportion changed as well over the course of the ischemic period (see Fig. 4B).

**Figure 4.**
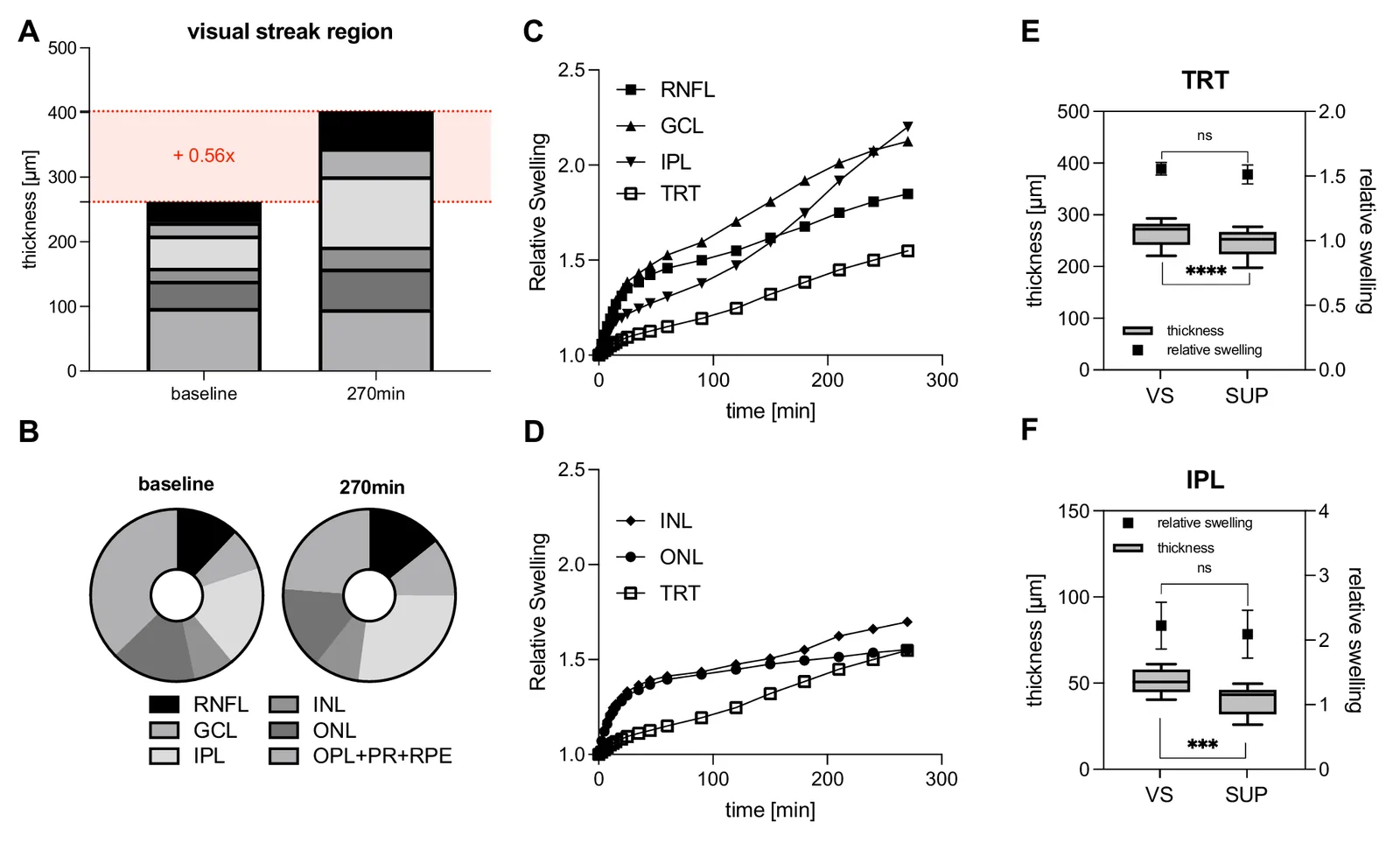
**Swelling behavior of the retinal layers and conservation across retinal regions.** Total retinal thickness increased about 1.56-fold in the visual streak area over the course of 270 minutes (**A**). Comparing the porcine retina after 270 minutes of ischemia with baseline revealed a remarkably changed composition of TRT (**B**). This was attributed by the severe differences in relative swelling rates of the different retinal layers (**C,** **D**, error bars are not shown for better clarity). Relative swelling of the retinal layers was conserved across different retinal regions, as exemplarily shown for TRT (**E**) and IPL (**F**). Although baseline thickness of both TRT and IPL showed a significant difference between visual streak and superior area, this significant difference did not occur when comparing relative swelling. GCL, ganglion cell layer; ONL, outer nuclear layer; OPL, outer plexiform layer; PR, photoreceptor layer; RPE, retinal pigment epithelium layer; SUP, superior retinal region; TRT, total retinal thickness; VS, visual streak.

These changes can be attributed to the differences in the relative swelling rates of the respective layers. This relative increase of layer thickness after 270 minutes of ischemia ranged from 1.53 ± 0.21-fold for the ONL to 2.21 ± 0.36-fold for the IPL in the VS. Including the superior and inferior region of the porcine retina, which supports this individual swelling behavior of the respective layers. The RNFL, IPL, and GCL present a more pronounced swelling than the INL and ONL (Figs. 4C, 4D). Within the early phase of ischemia, RNFL and GCL showed an initial rapid increase in thickness, after approximately 1 to 2 hours, increase in TRT was mostly driven by continuous swelling of the IPL. The outer retina including PR layer, RPE, and OPL presented an almost unchanged thickness (see Fig. 4A).

### Relative Swelling Is Conserved Across Different Regions of the Retina

TRT and individual layer thickness, as well as the composition of the TRT differs between the various regions of the porcine posterior pole, as observed in humans. When comparing the relative swelling of the retina, this effect is, however, abolished: whereas TRT differs significantly between the VS and the superior region (SUP) at baseline (265 ± 24 µm vs. 245 ± 26 µm, *P* < 0.0001), no significant differences occurred between the relative swelling rates after 270 minutes (*P* = 0.08; Fig. 4E).

This effect can be attributed to the swelling rates of the different retinal layers being conserved over the two different retinal regions, as exemplarily shown for the IPL (thickness at baseline VS versus SUP: 51.3 ± 7.1 µm vs. 40.3 ± 8.4 µm, *P* = 0.0006, swelling rate after 270 minutes VS versus SUP: *P* = 0.196; Fig. 4F).

## Discussion

In our study, we, for the first time, characterized the effect of ischemia in the porcine retina separately for each retinal layer in different regions of the porcine retina with precise and ultra-early granular time-resolution. Interestingly, we found that immediately after ischemia, certain inner layers of the retina showed a near linear swelling behavior. In the present study, baseline data of the perfused retina was available to also allow direct and relative comparison of retinal swelling, a parameter specifically interesting for translating and comparing our results to clinical observations. Additionally, this study evaluated the time course of the loss of neurophysiological activity of the neuroretina after ischemia, showing a complete loss of ERG-response as early as 2.5 minutes, highlighting the limited ischemic tolerance of neuroretinal tissue.

In retinal research, the pig model is a valuable tool to preclinically characterize diseases and to evaluate possible treatment.^24^^–^^28^ The porcine eye hereby shows significant similarities to the human eye especially regarding size,^29^ intraocular pressure,^30^ retinal ultrastructure,^31^ and chorioretinal vascularization.^32^^,^^33^ Cross-species conclusions and translation of research findings into clinical practice, as we aimed in this study for CRAO, are therefore feasible and viable. Despite pigs showing differences in the regional organization of the porcine posterior pole,^34^ they present the best-suited model for the investigation of vascular chorioretinal diseases aside from the nonhuman primate.

Objective determination of ischemia onset through OCT-based biomarkers was previously proposed in the light of time-critical treatment of CRAO. Although recent evidence has questioned the benefit-risk profile specifically for systemic thrombolysis, precise determination of ischemia onset remains highly relevant for understanding the temporal evolution of retinal injury and for evaluating future time-dependent therapeutic strategies. Given the high sensitivity of neuronal retinal tissue to ischemia, profound knowledge on temporal changes assessed by easy-to-access techniques like OCT may be beneficial when evaluating the therapeutic window, efficacy, and safety of emerging interventions.

Previous studies on OCT-biomarkers in CRAO largely followed two approaches: explanted porcine cadaver eyes and clinical data from humans suffering from CRAO.^20^^–^^22^ In an animal study by Ochakovski and colleagues, the authors specifically focused on the increase in absolute thickness and compared it to human data.^20^ Although they importantly characterized the linear manner of retinal swelling for the whole retina in a timeframe between approximately 1 to 9 hours after ischemia,^20^ the image quality in this study did not allow single layer characterization. Moreover, study designs using porcine cadaver eyes present substantial limitations, which we sought to address with the here presented study. Because the removal process of the eyes from the orbita of the porcine cadaver requires axotomy and changes intraocular pressure, an influence of this protocol on the study's results is considered.^35^^,^^36^ Furthermore, physiological retinal vasculature and perfusion before the ischemia cannot be assumed with certainty, especially because some pig breeds frequently suffer from cardiovascular diseases.^37^ Most importantly when using explanted cadaver eyes, time of death is more of an estimate and baseline data are lacking. This does not allow the calculation of relative thickness increases, which are assumed to be more robust compared with absolute values being subject to substantial intraindividual differences.

Inter-species and inter-individual robustness of relative thickness increase was used by studies analyzing clinical data from patients with CRAO.^21^^,^^22^ Wenzel et al. introduced relative retinal thickness increase (RRTI), where the retinal thickness was compared between the affected eye and the unaffected fellow eye. They found RRTI to discriminate patients with an ischemia of 4.5 hours at a maximum from such with an ischemia longer than 4.5 hours with a high sensitivity and specificity,^21^ results later strengthened by data on automatically measured retinal thickness.^22^ Both studies described RRTI between 20% and 30% after 4.5 hours.^21^^,^^22^ In our study, we report an increase of approximately 50% (see Fig. 2C, Fig. 4A). In cases of CRAO, only those retinal layers supplied by the intraretinal vasculature are affected from ischemia. Considering this, a theoretical increase of TRT of approximately 45% can be calculated in our model assuming exclusive ischemia of the inner retinal layers. This difference to the patients’ data can be attributed to the retrospective character of such clinical investigations likewise coming with substantial shortcomings. The most important aspect is the certainty of retinal ischemia onset. Because clinical data relies on how precisely the patient can report the first appearance of visual symptoms, this pivotal information is often uncertain.^38^^,^^39^ In addition, vascular and ocular co-pathologies may impair the validity of the results. Further, the temporal course of such clinical data on retinal swelling is far less granular, because the examination of patients with a high frequency is clinically not applicable. However, the relative retinal swelling observed across different retinal regions in our study is essentially within the range of clinical data. This strengthens the role and translational relevance of experimental porcine data for investigation of retinal swelling in ischemic diseases.

Furthermore, our model investigated retinal swelling layer-wise. Whereas the more pronounced swelling of the inner retina compared to the outer retina largely confirmed previous investigations, the high temporal granularity of our model combined with the high-resolution layer-wise analysis allowed a distinct characterization of layer-specific swelling kinetics within the early phase after ischemia. These distinct temporal swelling profiles may represent both their ultrastructural and metabolic heterogeneity.^40^ In particular, the rapid swelling of the two innermost retinal layers RNFL and GCL in the early phase after chorioretinal ischemia is consistent with the high metabolic demands of the retinal ganglion cells (RGCs) forming these layers.^41^^,^^42^ This retinal cell type specifically was previously characterized as highly vulnerable to ischemic stress,^43^^–^^45^ which may be attributed to a constant activity of these neurons, a substantial integration of signals from the outer retinal layers,^46^ and the unmyelinated nature of the axons of the RGCs forming the RNFL, requiring continuous axonal depolarization in absence of saltatory conduction.^47^ In combination, these properties contribute to a high metabolic demand^48^ leading to the formation of cytotoxic edema immediately after ischemia onset. In contrast, the IPL is a complex meshwork of synaptic contacts und supporting glial cells.^49^ Because the main metabolic activity of the RGCs and retinal bipolar cells (RBCs) forming synapses in the IPL is concentrated in their somata in the GCL and INL, as well as in the CNS-directional axons of the RGCs in the RNFL, edema formation in the IPL is likely delayed compared with the GCL and RNFL. However, due to the large membrane surface area resulting from extensive dendritic branching, the progressive breakdown of cellular membrane integrity over the course of ischemia has a particularly pronounced swelling effect. The high density of synaptic contacts with limited extracellular space could further enhance IPL swelling.

This layer-specific temporal swelling signature revealed by the novel combination of time- and layer-wise analysis in this model suggests a representation of cell-level heterogeneity in clinically measurable differences. This is of particular interest in cases where impairments of the retinal microvasculature cause layer-specific ischemia in the macular region resulting in conditions like paracentral acute middle maculopathy (PAMM)^50^ and acute macular neuroretinopathy (AMN).^51^ OCT imaging in these diseases often reveal formation of hyper-reflective edema in the INL (PAMM) or the OPL (AMN).^52^ Estimating the age of these lesions could improve future patient management.

The conservation of these layer-specific kinetics across different regions in turn may further provide valuable data for future retinal ischemia studies in the porcine model, as occlusive vascular retinal diseases were previously modeled in pigs using endovascular^53^^–^^55^ and embolization^56^^,^^57^ techniques. Given that edema formation is independent of location, future studies may investigate morphological changes within specific retinal layers at their thickest points without compromising the validity of the findings or the comparability of the data. In addition, porcine eyes, in some cases, have multiple ciliary arteries supplying different areas of the retina^55^ and the adhesion of an embolus along the course of a vessel is difficult to predict,^57^ our findings on layer-specific and cross-regional behavior of the porcine retina upon ischemia further serves as a valuable data basis for future experimental work in respective porcine animal disease models.

Apart from the ischemic behavior of the retinal layers, pronounced changes in the morphology of the choroidea appeared. Interestingly, an immediate complete collapse of the choroidea after induction of cardiac arrest was observed. This suggests that venous pressure plays a substantially greater role in maintaining choroidal shape than the surrounding connective tissue structure. This is especially important because explanted porcine eyes are frequently used in wet-lab testing of new surgical techniques in ophthalmology. The immediate collapse of the choroidea hereby sets substantial limitations to the use of porcine cadaver eyes in ex vivo studies regarding the safety and feasibility of suprachoroidal injection techniques, an emerging drug delivery route for the posterior segment.^58^

### Limitations

Some limitations of this study need to be considered. First, chorioretinal ischemia was induced by complete cardiac arrest of the animals which limits the applicability of the model to CRAO. On the one hand, conditions of CRAO are only partly mimicked, because, in these cases, choroidal perfusion and vitreous humor circulation is still intact, which can influence post-ischemic behavior and possibly alleviate hypoperfusion damage. On the other hand, some residual intraretinal perfusion can be observed in many cases of CRAO, also known as incomplete occlusion, which can also be addressed only partly in a chorioretinal ischemia model based on cardiac arrest. This needs to be considered upon translation of the findings into clinical practice. Second, although the eye was left inside the orbital cavity of the animals and the animals were heated to simulated body temperature, this experimental setup can only insufficiently cover the conditions within a living animal or human. Third, the time course of the study is limited to 270 minutes, as we referred to the proposed window of lysis for ischemic stroke and CRAO. However, this does not cover the late ischemia-related changes in chorioretinal morphology, as seen in patients presenting beyond 4.5 hours after ischemia onset, especially in cases when symptoms occurred upon awakening.

## Conclusions

OCT biomarkers based on retinal swelling are time-dependent, layer-specific, and conserved across regions. They may therefore help to estimate ischemia onset in ischemic retinal diseases. Furthermore, this study provides a data basis for future translational studies on ischemic chorioretinal diseases.

## Supplementary Material

Supplement 1
