# Supplementary material for: Time-, Location-, and Layer-Specific Chorioretinal Biomarkers in a Translational Large-Animal Porcine Model of Chorioretinal Ischemia
Source: Invest Ophthalmol Vis Sci. 2026 Oct 5;67(12):8. doi: 10.1167/iovs.67.12.8 (PMC13657118; doi:10.1167/iovs.67.12.8)
Supplement: Supplementary file 1 [file iovs-67-12-8_s001.pdf]

Supplementary Material

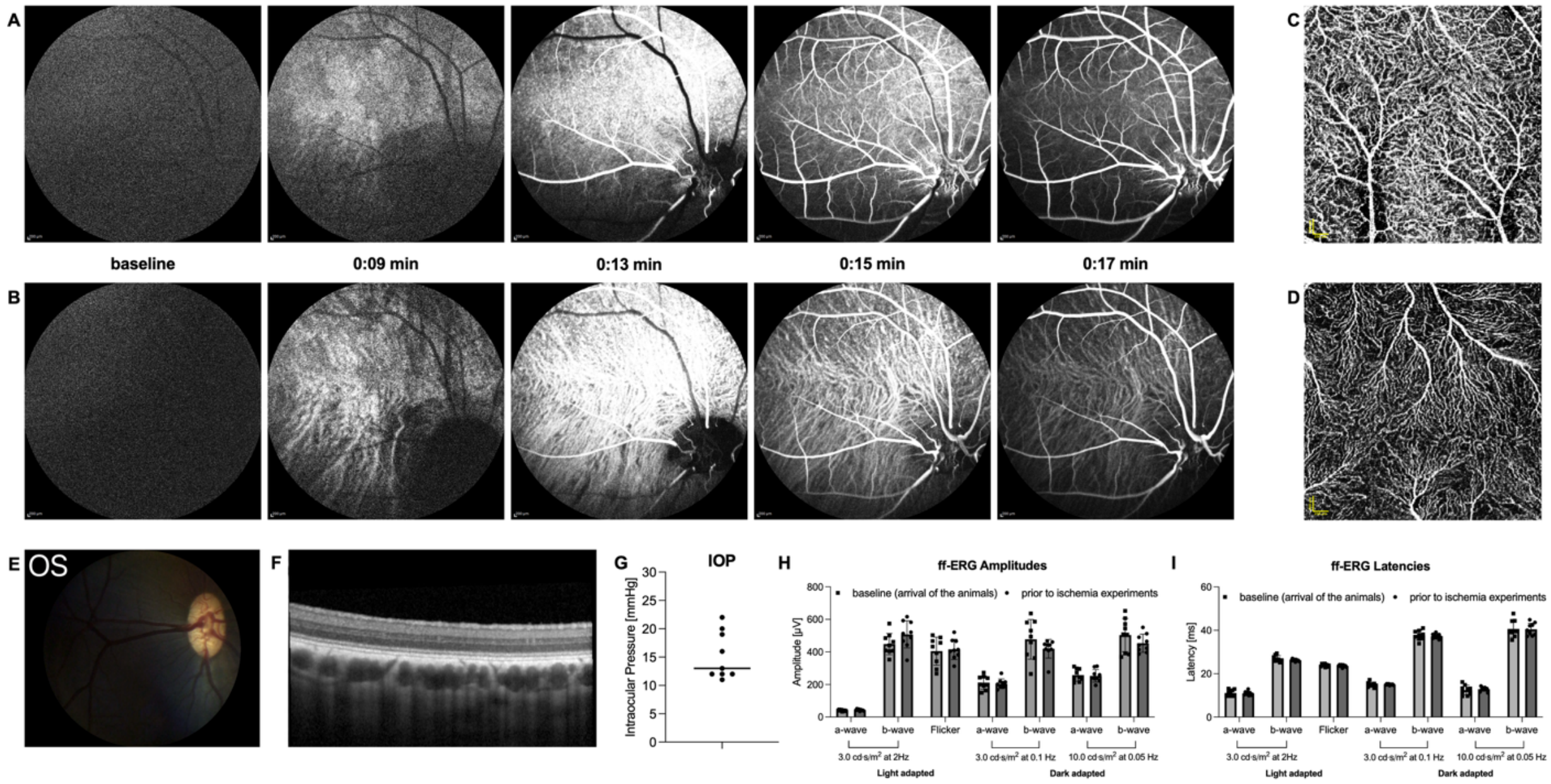

Supplementary Figure S1: Health status assessment of the eyes included in the ischemia experiments and chorioretinal angiography prior to induction of ischemia. Pre-ischemic angiography was performed to ensure physiological vascularization and perfusion of the porcine retina. Normal filling of the retinal and choroidal macrovasculature and absence of malperfusion or vascular hyperpermeability was confirmed by means of FA **(A)** and ICGA **(B).** Configuration of the intraretinal microvasculature was assessed using OCT-A with the superficial **(C)** and the deep **(D)** vascular complex showing normal intraretinal capillary network. Aside from chorioretinal vascularity, the health status of the included eyes was further ensured by multimodal ophthalmic examination. Representative fundus photographs **(E)** and OCT-scans **(F)** showed a healthy posterior segment morphology and retinal ultrastructure. **(G)** The intraocular pressure was within normal limits in all included eyes. **(H-I)** Amplitudes and latencies of 4 different performed photopic and mesopic ERG testing protocols revealed normal values prior to ischemia experiments, especially when compared to the values at arrival of the animals in the facility 6 weeks prior to the ischemia experiments. FA, ICGA, OCT, OCT-A and fundus images are shown for one representative animal.

Abbreviations: FA – fluorescein angiography, **ICGA** – indocyanine green angiography, **OCT** – optical coherence tomography, **OCT-A** – optical coherence tomography angiography

Supplementary Table S1: Parameters of the two performed ERG-tests

Name	Conditions	Flash		Response
		Intensity [cd/m^2]	Frequency [Hz]	
LA_3.0	photopic	3.0	2	mixed (cones + rods)
Flicker	photopic	3.0	28.3	cone-isolated

Supplementary Table S2: Thickness of the respective retinal layers at baseline and 270 minutes post mortem in the 3 different regions

layer	baseline			270 minutes		
	Mean [µm]	SD [µm]	N	Mean [µm]	SD [µm]	N
**Visual Streak Region (VS)**						
RNFL	31.67	4.34	10	58.10	6.16	9
GCL	21.12	2.17	10	45.55	6.78	9
IPL	51.33	7.11	10	110.37	10.62	9
INL	20.37	3.71	10	35.37	8.75	9
ONL	42.47	7.13	10	63.75	8.53	9
OPL+PR+RPE	98.32	11.90	10	94.09	18.98	9
TRT	265.29	24.25	10	407.21	29.82	9
**Superior Region (SUP)**						
RNFL	28.04	3.08	10	51.90	6.39	9
GCL	19.51	3.90	10	40.20	7.12	9
IPL	40.27	8.35	10	81.87	16.76	9
INL	20.16	4.01	10	33.13	7.71	9
ONL	45.76	9.61	10	66.77	8.75	9
OPL+PR+RPE	92.62	15.07	10	93.62	20.83	9
TRT	246.36	25.87	10	367.49	39.80	9
**Inferior Region (VS)**						
RNFL	35.02	4.49	10	65.46	8.58	9
GCL	20.96	2.32	10	42.71	7.28	9
IPL	46.03	8.09	10	101.29	18.412	9
INL	21.89	4.04	10	35.21	8.73	9
ONL	41.77	7.15	10	68.17	8.52	9
OPL+PR+RPE	93.63	10.52	10	89.42	15.06	9
TRT	259.31	25.53	10	402.27	34.42	9
